# Effect of F-18 Fluorodeoxyglucose Uptake by Bone Marrow on the Prognosis of Head and Neck Squamous Cell Carcinoma

**DOI:** 10.3390/jcm8081169

**Published:** 2019-08-04

**Authors:** Jeong Won Lee, Myung Jin Ban, Jae Hong Park, Sang Mi Lee

**Affiliations:** 1 Department of Nuclear Medicine, Catholic Kwandong University College of Medicine, International St. Mary’s Hospital, Incheon 22711, Korea; 2 Department of Otorhinolaryngology-Head and Neck Surgery, Soonchunhyang University Cheonan Hospital, Cheonan 31151, Korea; 3 Department of Nuclear Medicine, Soonchunhyang University Cheonan Hospital, Cheonan 31151, Korea

**Keywords:** head and neck cancer, fluorodeoxyglucose F-18, positron emission tomography, bone marrow, prognosis

## Abstract

The purpose of this study was to assess the relationship between F-18 fluorodeoxyglucose (FDG) uptake in bone marrow (BM) on positron emission tomography/computed tomography (PET/CT) and survival in patients with head and neck squamous cell carcinoma (HNSCC). We retrospectively enrolled 157 HNSCC patients who underwent staging FDG PET/CT and subsequent treatment. On PET/CT, primary tumor metabolic characteristics, mean FDG uptake of BM (BM SUV), and BM-to-liver uptake ratio (BLR) were measured. The prognostic significance of FDG uptake of BM for predicting disease progression-free survival and distant failure-free survival was assessed using a Cox proportional hazards regression model. In univariate analysis for disease progression-free survival, increased BM SUV and BLR were associated with poor survival. In multivariate analysis, BLR (*p* = 0.044; hazard ratio, 1.96), TNM stage (*p* = 0.014; hazard ratio, 2.87) and maximum FDG uptake of the primary tumor (*p* = 0.046; hazard ratio, 2.38) were independently associated with disease progression-free survival. For distant failure-free survival, BLR, TNM stage, tumor size, and metabolic parameters of the primary tumor showed prognostic significance in univariate analysis. However, none of the variables showed significance in multivariate analysis. FDG uptake of BM in HNSCC patients might be a significant predictor for disease progression-free survival. Further studies with large patient population are needed to validate the results.

## 1. Introduction

Chronic inflammation induced by infection is strongly correlated with the development of cancer. For instance, human papilloma virus infection is associated with head and neck cancer, Epstein-Barr virus infection with nasopharyngeal cancer, and hepatitis virus infection with hepatocellular carcinoma. In recent decades, evidence has increasingly suggested that the host inflammatory response mediated via pro-inflammatory cytokines and immune cells such as neutrophils and tumor-activated platelet is involved in the initiation, progression, and metastasis of cancer cells [1,2]. Inflammation has even been considered the seventh hallmark of cancer, in addition to sustaining proliferation, evading growth suppression, activating invasion and metastasis, enabling immortality, inducing angiogenesis, and resisting cell death [3]. In a clinical setting, serum inflammatory markers, including the neutrophil-to-lymphocyte ratio (NLR), platelet-to-lymphocyte ratio (PLR), and C-reactive protein (CRP) level, have commonly been used to estimate the degree of inflammatory reaction of the patient [4,5]. Because of the significant link between the inflammatory reaction and tumor progression, NLR, PLR, and CRP have been found to have significant prognostic value for predicting survival in diverse cancers including head and neck squamous cell carcinoma (HNSCC) [5,6,7,8]. Based on these results, biomarkers for a systemic inflammatory response are considered to be a tool for predicting clinical outcomes in malignant diseases [5,6,7,8].

In addition to serum inflammatory markers, several recent studies have attempted to find an imaging biomarker for the systemic inflammatory response by using F-18 fluorodeoxyglucose (FDG) positron emission tomography/computed tomography (PET/CT) [9,10,11]. FDG PET/CT is a diagnostic imaging modality that has been widely used for staging, assessment of treatment response, detecting recurrence, and predicting prognosis in HNSCC patients [12,13,14,15]. In patients with malignant diseases, including HNSCC, bone marrow (BM) often shows increased FDG uptake in staging PET/CT, while only minimal-to-mild FDG uptake in the BM is observed in healthy subjects [16,17,18,19]. FDG uptake by the BM indicates the glucose metabolism of immune cells and demonstrates significant association with serum inflammatory markers, suggesting that the FDG uptake of the BM could be used as an imaging biomarker of the systemic inflammatory response [11,20]. The mean standardized uptake value (SUV) of the BM (BM SUV) has typically been used to represent FDG uptake of the BM [9,21], but the BM-to-normal liver uptake ratio (BLR) has also been suggested as a parameter for estimating FDG uptake of the BM because correction of FDG uptake by normal liver tissue has been shown to reduce inter-individual variation of FDG uptake of the BM [16]. In clinical studies of small cell lung cancer, non-small cell lung cancer, gastric cancer, lymphoma, uterine cervical cancer, colorectal cancer, and breast cancer, both FDG uptake of the BM and the BLR was significantly associated with clinical outcomes [9,10,11,19,20,21,22,23]. Furthermore, a recent meta-analysis has demonstrated that patients with low FDG uptake of the BM have better event-free survival and overall survival than those with high uptake values [24]. However, for HNSCC, only a single retrospective study of 35 patients has evaluated the clinical implications of FDG uptake of the BM on PET/CT to date, and this study had constraints that limited clinical significance of FDG uptake of the BM [18].

The objective of this study was to investigate the relationship of FDG uptake of the BM with serum inflammatory markers and tumor factors and to assess the prognostic value of FDG uptake of the BM for predicting disease progression-free survival and distant failure-free survival in patients with HNSCC.

## 2. Materials and Methods

### 2.1. Patient Population

The Institutional Review Board of Soonchunhyang University approved this retrospective study and the requirement to obtain informed consent was waived. All procedures in this study were performed in accordance with the Declaration of Helsinki. Medical records of all patients who were histopathologically diagnosed with HNSCC in our hospital between January 2011 and December 2016 were retrospectively reviewed. Among these patients, we finally enrolled 157 patients based on the following inclusion criteria:

- Patients with HNSCC who had undergone FDG PET/CT for initial staging work-up
- Patients who had no evidence of distant metastasis on staging imaging examinations
- Patients who had received any type of curative or palliative treatment after staging work-up

The exclusion criteria for the study were as follows:

- Patients who had distant lymph node or organ metastasis on staging imaging studies
- Patients who had received any treatment before PET/CT scan
- Patients who underwent only supportive care without any palliative treatment
- Patients who had coexisting acute inflammatory disease at the time of staging work-up
- Patients who were diagnosed with chronic liver disease
- Patients who had a previous history of another malignancy or BM disease
- Patients who were not followed up for 12 months after the initial treatment without disease progression

### 2.2. Staging Work-Up and Follow-Up

All enrolled patients had undergone staging examinations consisting of blood tests, direct laryngoscopy, planar chest radiographs, contrast-enhanced CT, and magnetic resonance imaging of the head and neck area, as well as FDG PET/CT. For hematologic parameters, blood cell counts including differential counts of white blood cells and hemoglobin levels were determined. For serum inflammatory markers, serum CRP was measured and NLR and PLR were calculated based on the results of differential counts in the blood test. The NLR was defined as the neutrophil counts divided by the lymphocyte counts and the PLR was defined as the platelet counts dived by the lymphocyte counts. After pretreatment work-up, the clinical T and N stages of the patients were assessed according to the seventh edition of the American Joint Committee on Cancer Staging Guidelines. Subsequently, patients received curative or palliative treatment including surgery, chemotherapy, and radiotherapy based on their clinical stage and condition within four weeks after FDG PET/CT. After the initial treatment, all patients underwent regular clinical follow-up including blood tests, direct laryngoscopy, and contrast-enhanced CT every 3–6 months. Patients were categorized into two groups according to the pattern of disease progression during the follow-up: loco-regional failure (disease progression of a known cancer lesion and/or recurrence in the primary site and/or bilateral neck area) and distant failure (distant lymph node and organ metastasis irrespective of loco-regional failure).

### 2.3. FDG PET/CT Imaging

FDG PET/CT scans were performed on a dedicated PET/CT scanner (Gemini, Philips, Milpitas, CA, USA and Biograph mCT 128 scanner, Siemens Healthcare, Knoxville, TN, USA) from the skull base to the proximal third of the thigh. All patients fasted for at least six hours before FDG PET/CT and blood glucose levels were less than 200 mg/dL before administration of FDG. One hour prior to scanning, a dose of approximately 5.18 MBq/kg of FDG was intravenously injected for the Gemini and approximately 4.07 MBq/kg for the Biograph mCT 128 scanner. First, a CT scan was performed without contrast enhancement for attenuation correction at 80 mA and 140 kV_p_ for the Gemini and at 100 mA and 120 kV_p_ for the Biograph mCT 128 scanner. Subsequently, a PET scan was performed at 2.5 min per bed position for the Gemini and at 1.5 min per bed position for the Biograph mCT 128 scanner in the three-dimensional acquisition mode. PET images were reconstructed by using an iterative reconstruction algorithm with attenuation correction.

### 2.4. Imaging Analysis

PET/CT images of the enrolled patients were retrospectively assessed by two board-certified nuclear medicine physicians who were blinded to the clinical outcomes. Discrepancies between the readers were resolved by discussion to consensus. Initially, three metabolic PET/CT parameters of the primary cancer lesion (maximum SUV, metabolic tumor volume, and total lesion glycolysis) were measured. Each primary tumor lesion was examined using a spheroid-shaped volume-of-interest that included the entire cancer lesion in the axial, coronary, and sagittal planes. The maximum SUV of the primary tumor was calculated using the formula: (decay-corrected activity/tissue volume)/(injected dose/body weight). The boundaries of voxels with an SUV of 2.50 or greater were automatically produced within volume-of-interest of the primary tumor. The total volume of voxels within the boundaries was measured and defined as the metabolic tumor volume. The mean SUV of those voxels was also measured. Total lesion glycolysis of the primary cancer lesion was calculated by multiplying the metabolic tumor volume with the mean SUV. Subsequently, PET/CT parameters of the BM including BM SUV and BLR were measured. Spheroid-shaped volume-of-interests were drawn over the vertebral body of each of the six vertebrae in the thoracic and lumbar spine. Spines that showed compression fracture, severe osteoarthritic change, or post-operative change of spinal surgery were excluded from the measurement. Within each volume-of-interest in the vertebral body, an automatic isocontour set at an SUV of 75% of the maximum SUV was automatically generated and the mean SUV of voxels within the isocontour was calculated. This method of using the cut-off value of 75% of the maximum SUV had shown good reproducibility for measuring FDG uptake of the BM in a previous study [20]. The mean SUV of the volume-of-interests in the six selected vertebrae was calculated and defined as BM SUV. A 2 cm-sized spheroidal volume-of-interest was drawn in the right lobe of the liver and the mean SUV of the normal liver tissue was also measured. Using BM SUV and the mean SUV of the normal liver tissue, the BLR was calculated for each patient.

### 2.5. Statistical Analysis

Spearman’s rank correlation and Mann-Whitney U tests were performed to determine the relationship of FDG uptake of the BM with hematologic parameters, serum inflammatory markers, tumor size, PET/CT parameters of primary tumor, and clinical stage. The association of FDG uptake of the BM, PET/CT parameters, and clinical factors with disease progression-free survival and distant failure-free survival was investigated using a Cox proportional hazards regression model for univariate and multivariate analyses. The survival time was defined as the time from the date of FDG PET/CT to the date of the detection of disease progression. For patients without disease progression, the follow-up was censored at the day of the last follow-up visit. Continuous variables in the survival analysis were dichotomized according to specific optimal cut-off values determined by receiver operating characteristic curve analysis. Survival curves for disease progression-free survival and distant failure-free survival were estimated using the Kaplan-Meier method and survival differences between the groups were assessed using the log-rank test. Statistical analyses were performed with MedCalc Statistical Software, version 19.0.3 (MedCalc Software bvba, Ostend, Belgium), in which *p*-values < 0.05 were considered statistically significant.

## 3. Results

### 3.1. Clinical Characteristics of Patients

**Figure 1:**
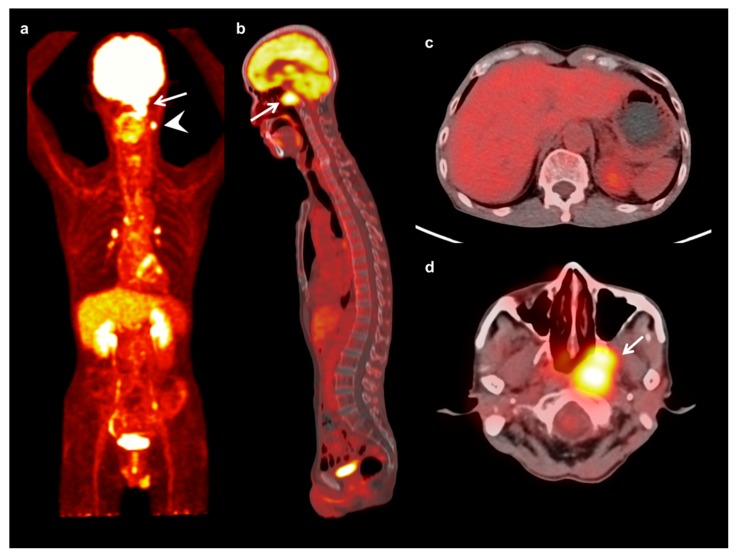
Maximum intensity projection (**a**) and sagittal (**b**) FDG PET images and fused transaxial FDG PET/CT images (**c**,**d**) of a 64-year-old man with nasopharyngeal cancer. A 3 cm-sized mass in the left nasopharynx showed intensely increased FDG uptake (arrow) (maximum SUV 9.53, metabolic tumor volume 28.83 cm^3^, and total lesion glycolysis 132.62 g) with ipsilateral neck lymph node metastases (arrowhead). Serum CRP level, NLR, and PLR of the patients were 3.02 mg/dL, 2.53, and 212.38, respectively. On FDG PET/CT, only a mild degree of FDG uptake was shown in the bone marrow (BM SUV of 1.13 and BLR of 0.64). The patient was clinically diagnosed with T3N1, stage III, and underwent concurrent chemoradiotherapy. The patient has not experienced cancer progression during 48.8 months of follow-up.

The clinical characteristics of the 157 enrolled patients are summarized in Table 1. The mean age of the patients was 61 ± 11 years. More than 80% of the patients were male. The most common site of the primary cancer lesion was the oropharynx and oral cavity (45.9%), followed by the hypopharynx (39.5%) and nasopharynx (14.6%; Figure 1). Overall, 74 patients (47.1%) had regional lymph node metastasis on staging work-up and 55 patients (35.0%) were diagnosed as stage IV. FDG PET/CT revealed a metabolic tumor volume of 0.0 cm^3^ in nine patients (5.7%) and a subsequent total lesion glycolysis of 0.0 g because the maximum SUV of primary tumor was less than 2.50. Radical surgery alone or surgery with adjuvant therapy was performed in 95 patients (60.5%), and 53 patients (33.8%) received concurrent chemoradiotherapy. The mean follow-up period in the enrolled patients was 25.6 months (range 0.3–84.5 months). During the follow-up, 48 patients (30.6%) experienced disease progression, including 31 patients (19.7%) with loco-regional failure and 17 (10.8%) with distant failure, and 13 patients (8.3%) died.

### 3.2. FDG Uptake of BM

**Figure 2:**
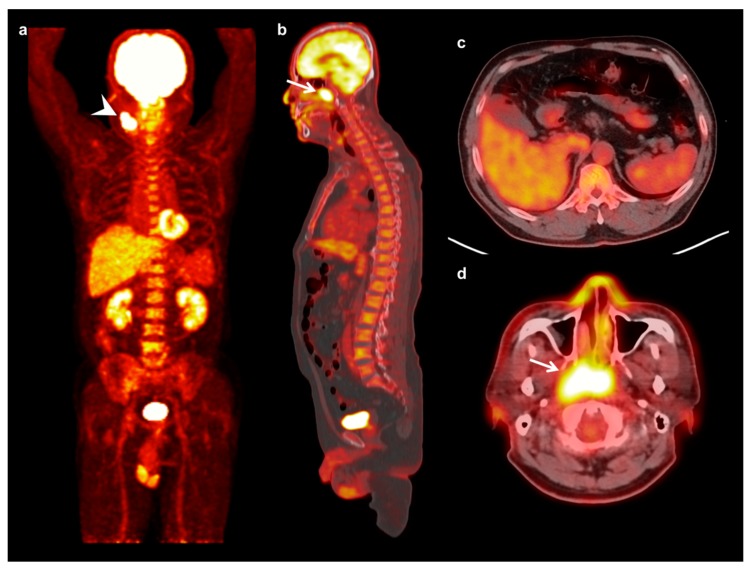
Maximum intensity projection (**a**) and sagittal (**b**) FDG PET images and fused transaxial FDG PET/CT images (**c**,**d**) of a 57-year-old man with nasopharyngeal cancer. A 5.3 cm-sized mass in the right nasopharynx showed intensely increased FDG uptake (arrow) (maximum SUV 12.61, metabolic tumor volume 44.42 cm^3^, and total lesion glycolysis 237.65 g) with ipsilateral neck lymph node metastases (arrowhead). Serum CRP level, NLR, and PLR of the patients were 6.38 mg/dL, 5.10, and 243.01, respectively. On FDG PET/CT, markedly increased FDG uptake was shown in the bone marrow (BM SUV of 2.37 and BLR of 1.03). The patient was clinically diagnosed with T3N2, stage III, and underwent concurrent chemoradiotherapy. The cancer lesion progressed at 9.1 months after treatment.

Among the 157 patients enrolled, BM SUV in 27 patients (17.2%) was higher than the mean SUV of the normal liver (BLR > 1.0; Figure 2). In correlation analysis of BM SUV and BLR with hematologic parameters, serum inflammatory markers, and tumor factors, both BM SUV and BLR showed significant positive correlations with serum CRP (*p* = 0.045, r = 0.148 for BM SUV; *p* = 0.009, r = 0.208 for BLR), NLR (*p* = 0.014, r = 0.195 for BM SUV; *p* = 0.015, r = 0.195 for BLR), tumor size (*p* = 0.014, r = 0.196 for BM SUV; *p* = 0.001, r = 0.270 for BLR), maximum SUV (*p* < 0.001, r = 0.282 for BM SUV; *p* < 0.001, r = 0.314 for BLR), metabolic tumor volume (*p* < 0.001, r = 0.332 for BM SUV; *p* < 0.001, r = 0.389 for BLR), and total lesion glycolysis (*p* < 0.001, r = 0.321 for BM SUV; *p* = 0.005, r = 0.377 for BLR). BLR also showed a significant positive correlation with PLR (*p* = 0.017, r = 0.191). In contrast, the white blood cell count (*p* = 0.829 for BM SUV; *p* = 0.233 for BLR) and hemoglobin level (*p* = 0.060 for BM SUV; *p* = 0.186 for BLR) had no significant correlation with either BM SUV or BLR. Results for the relationship between clinical stage and FDG uptake of BM demonstrated that patients with T3T4 stage, N1–N3 stage, and stage III–IV had significantly higher BM SUV and BLR values than those with T1T2 stage, N0 stage, and stage I–II, respectively (*p* < 0.05; Table 2).

### 3.3. Disease Progression-Free Survival Analysis

**Figure 3:**
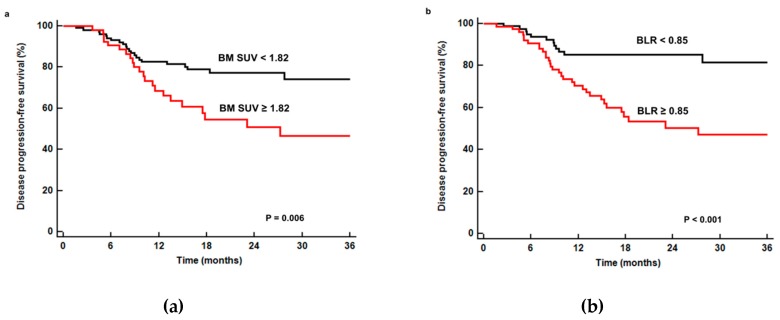
Cumulative disease progression-free survival curves according to BM SUV (**a**) and BLR (**b**). Patients with low BM SUV (<1.82) and BLR (<0.85) showed significantly better disease progression-free survival (*p* = 0.006 for BM SUV and *p* < 0.001 for BLR) than those with high BM SUV (≥1.82) and BLR (≥0.85), respectively.

The prognostic values of age, sex, smoking, T stage, N stage, TNM stage, tumor size, serum CRP level, NLR, PLR, maximum SUV, metabolic tumor volume, total lesion glycolysis, BM SUV, and BLR were assessed in the survival analysis. The optimal cut-off values for continuous variables were 60 years for age, 3.0 cm for tumor size, 5.0 mg/dL for serum CRP level, 2.75 for NLR, 201.00 for PLR, 6.60 for maximum SUV, 8.50 cm^3^ for metabolic tumor volume, 32.50 g for total lesion glycolysis, 1.82 for BM SUV, and 0.85 for BLR. In univariate analysis, T stage, N stage, TNM stage, tumor size, serum CRP level, PLR, maximum SUV, metabolic tumor volume, total lesion glycolysis, BM SUV, and BLR were significantly associated with disease progression-free survival (*p* < 0.05; Table 3). Patients with high FDG uptake of the BM showed worse survival than those with low FDG uptake; median disease progression-free survival was significantly shorter in patients with high BM SUV (27.2 months versus 64.9 months, *p* = 0.006; Figure 3a) and high BLR (23.1 months versus 64.9 months, *p* < 0.001; Figure 3b).

Among the variables that showed statistical significance in univariate analysis, TNM stage, tumor size, serum CRP level, PLR, maximum SUV, total lesion glycolysis, BM SUV, and BLR were selected for multivariate analysis (Table 3). Because there was a significant correlation between BM SUV and BLR (*p* < 0.001, r = 0.664), they were assessed in separate models. In multivariate analysis, TNM stage, PLR, and maximum SUV significantly predicted disease progression-free survival in the model containing BM SUV (*p* < 0.05), whereas BM SUV failed to show statistical significance (*p* = 0.072). In the model containing BLR, the significant determinants were TNM stage, maximum SUV, and BLR (*p* < 0.05; Table 3).

### 3.4. Distant Failure-Free Survival Analysis

**Figure 4:**
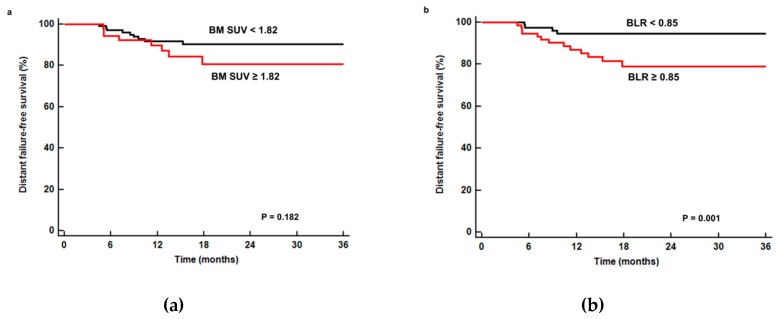
Cumulative distant failure-free survival curves according to BM SUV (**a**) and BLR (**b**). Patients with low BLR (<0.85) showed significantly better distant failure-free survival (*p* = 0.001) than those with high BLR (≥0.85), while no significant difference of survival was shown between patients with low (<1.82) and high (≥1.82) BM SUV (*p* = 0.182).

We also evaluated whether variables that were included in disease progression-free survival analysis had prognostic value in distant failure-free survival. In univariate analysis, N stage, TNM stage, tumor size, maximum SUV, metabolic tumor volume, total lesion glycolysis, and BLR were significantly associated with distant failure-free survival (*p* < 0.05; Table 4), while BM SUV was not a significant factor (*p* = 0.189; Figure 4a). Patients with a low BLR showed significantly higher 1-year distant failure-free survival than those with a high BLR (97.4% versus 84.7%, *p* = 0.001; Figure 4b). Of the variables included in univariate analysis, TNM stage, tumor size, maximum SUV, TLG, and BLR were selected for multivariate analysis. However, in multivariate analysis, none of these variables showed significant association with distant failure-free survival (*p* > 0.05; Table 4).

## 4. Discussion

In the present study, we measured FDG uptake of the BM in PET/CT images obtained in patients diagnosed with HNSCC and showed that FDG uptake of the BM was significantly associated with serum inflammatory markers, FDG PET parameters of the primary tumor, clinical stage, and disease progression-free survival. Among the various hematopoietic and stromal cells in the BM, FDG uptake of the BM is known to mainly reflect metabolic activity of granulocyte progenitors and is correlated with myeloid hyperplasia [25,26]. In previous studies, patients diagnosed with malignant diseases could show increased FDG uptake of the BM without malignant involvement, demonstrating higher FDG uptake of the BM in cancer patients than in patients with benign diseases and in healthy normal subjects [17,19,27]. Therefore, BM hypermetabolism in malignant diseases is considered to be due to a systemic inflammatory response of the host to cancer cells [16,17,20]. This hypothesis is supported by previous studies that have consistently reported a significantly positive correlation between FDG uptake of the BM and serum inflammatory markers including white blood cell count, serum CRP level, NLR, and PLR in diverse malignant diseases, and was also shown in the present study [10,11,21,22]. Furthermore, the clinical stage and metabolic PET/CT parameters of the primary tumor also showed a significantly positive correlation with FDG uptake of BM. This might indicate an enhanced inflammatory response in patients with aggressive and advanced cancer, which is consistent with previous reports [9,10,21,27].

Given that the degree of inflammatory response is related to the prognosis in patients with HNSCC [6,28,29], we evaluated the prognostic value of BM SUV and BLR in the present study. The prognostic significance of BM SUV and BLR has previously been assessed in various cancers. Increased BM SUV has been associated with worse disease-free survival in breast cancer, colorectal cancer, small cell lung cancer, and non-Hodgkin’s lymphoma, and worse pelvic recurrence-free survival in cervical cancer [9,19,21,23,30]. On the other hand, BLR has been significantly associated with disease-free survival in cervical cancer, non-Hodgkin’s lymphoma, and gastric cancer, and with overall survival in malignant pleural mesothelioma and gastric cancer [10,19,22,31]. For non-small cell lung cancer, BLR consistently has shown significant association with disease-free survival but not with overall survival irrespective of treatment modalities [11,20]. In a recent meta-analysis of 10 studies including 1197 patients [24], the combined hazard ratio of FDG uptake of the BM was 1.75 (95% confidence interval, 1.45–2.11) in the event-free survival analysis and 1.40 (95% confidence interval, 1.13–1.73) in the overall survival analysis using a random effects model; this suggests a role for FDG uptake of the BM in stratifying the risk of tumor progression. Similarly to previous studies, the present study demonstrates poor disease progression-free survival of HNSCC in patients with high FDG uptake of the BM, with a corrected hazard ratio of 1.96 for the BLR in multivariate analysis. The results of our study might indicate that the BLR could also be used as an imaging parameter for estimating inflammatory responses in HNSCC patients. Recently, cancer-related inflammation has emerged as a potentially new cancer treatment target and several attempts have been made to utilize anti-inflammatory drugs for cancer prevention and management [32,33]. Based on the results of the present study, BLR might be useful as an imaging biomarker for selecting therapeutic candidates when targeting cancer-related inflammation in HNSCC.

In the literature, only one study with 35 retrospectively enrolled HNSCC patients has evaluated the relationship of FDG uptake of the BM with disease-free survival and overall survival to date [18]. Similarly to the present study, the study determined that BM SUV was significantly associated with disease-free survival [18]. However, the small sample size and relatively short follow-up period (median 12.5 months), as well as the large proportion of stage IV patients (63%) among the enrolled patients, may have limited the significance of those results. In contrast, we enrolled a larger number of patients with a relatively even distribution among the TNM stages. Furthermore, in our study, the BLR showed superior prognostic value for disease progression-free survival, suggesting that the BLR might be preferable over BM SUV for predicting clinical outcome in HNSCC patients, similarly to previous studies on cervical cancer, gastric cancer, and non-small cell lung cancer [10,11,20,22].

Distant failure is one of the major causes of morbidity and mortality in patients with HNSCC [34]. Patients who have manifested distant metastases after initial treatment have had a dismal prognosis with a mean survival period of 7.5 months after the diagnosis of distant failure [34]. Because systemic inflammatory response also plays a role in cancer metastasis [35,36], we further investigated the association between FDG uptake of the BM and distant failure-free survival. Although patients with a high BLR showed worse distant failure-free survival than those with a low BLR, both BM SUV and BLR failed to show statistical significance upon multivariate analysis. However, considering the small numbers of events and the heterogeneous patient population, a future study should aim to clarify the relationship between FDG uptake of the BM and risk of distant failure using a large patient population in a prospective trial.

The study had several limitations that remain to be addressed. First, the study was retrospectively performed in a single center and may therefore involve selection bias. Hence, further studies are required to validate the results of the present study. Second, because we enrolled patients with identical histopathology (squamous cell carcinoma) of the head and neck area, the enrolled patients had diverse sites of the primary tumor and the recurrence potential of tumors might vary according to the tumor sites [29]. Third, in patients with oropharyngeal cancer, differences in clinical characteristics and survival outcomes have been shown according to human papilloma virus status, and human papilloma virus status has been included in the oropharyngeal cancer staging system of the eighth edition of the American Joint Committee on Cancer Staging Guidelines [37]. However, because human papilloma virus status was determined in only a few of the enrolled patients, we did not perform FDG uptake analysis in the BM based on patients’ human papilloma virus status. Fourth, because of the small number of deceased patients during follow-up, the prognostic significance of FDG uptake of the BM for overall survival could not be assessed. Finally, further studies regarding the relationship of FDG uptake of the BM with serum cytokine levels and histopathological BM findings are needed to elucidate the mechanism of FDG uptake by the BM in HNSCC patients.

## 5. Conclusions

In conclusion, the BLR showed significant prognostic value for predicting disease progression-free survival in HNSCC patients. Patients with a high BLR showed significantly worse disease progression-free survival that those with a low BLR. In patients with HNSCC, the FDG uptake of the BM in PET/CT may be an imaging biomarker that reflects the degree of systemic inflammatory response and the risk of disease progression. Although there are several limitations to the general application of our results, the present study may form the basis for future studies seeking imaging biomarkers of inflammatory response in HNSCC patients.

## Figures and Tables

**Table 1:** Clinical characteristics of the patients (*n* = 157).

	Characteristics	Mean ± SD	Number (%)
Age (years)		61 ± 11	
Sex	Men		131 (83.4%)
	Women		26 (16.6%)
Smoking	Current or former		87 (55.4%)
	Never		70 (44.6%)
Tumor location	Nasopharynx		23 (14.6%)
	Oropharynx and oral cavity		72 (45.9%)
	Hypopharynx		62 (39.5%)
T stage	T1		57 (36.3%)
	T2		45 (28.7%)
	T3		26 (16.6%)
	T4		29 (18.5%)
N stage	N0		83 (52.9%)
	N1		27 (17.2%)
	N2		41 (26.1%)
	N3		6 (3.8%)
TNM stage	Stage I		44 (28.0%)
	Stage II		23 (14.6%)
	Stage III		35 (22.3%)
	Stage IV		55 (35.0%)
Blood tests	White blood cell count (×10^12^ cells/L)	7.48 ± 3.04	
	Absolute neutrophil count (×10^12^ cells/L)	4.63 ± 2.69	
	Hemoglobin (g/dL)	13.1 ± 2.2	
	CRP (mg/dL)	14.35 ± 33.52	
	NLR	3.21 ± 3.72	
	PLR	161.88 ± 92.30	
Tumor size (cm)		2.5 ± 1.5	
PET/CT parameters of primary tumor	Maximum SUV	9.34 ± 5.36	
	MTV (cm^3^)	14.23 ± 21.71	
	TLG (g)	80.92 ± 159.51	
Mean FDG uptake of normal liver tissue		2.06 ± 0.32	
PET/CT parameters of BM	BM SUV	1.73 ± 0.36	
	BLR	0.85 ± 0.18	
Treatment	Surgery		50 (31.8%)
	Surgery + chemotherapy, radiotherapy, or chemoradiotherapy		45 (28.7%)
	Concurrent chemoradiotherapy		53 (33.8%)
	Radiotherapy alone		8 (5.1%)
	Chemotherapy alone		1 (0.6%)

Legend: CRP, C-reactive protein; NLR, neutrophil-to-lymphocyte ratio; PLR, platelet-to-lymphocyte ratio; PET/CT, positron emission tomography/computed tomography; SUV, standardized uptake value; MTV, metabolic tumor volume; TLG, total lesion glycolysis; FDG, F-18 fluorodeoxyglucose; BM, bone marrow; BM SUV, mean FDG uptake of bone marrow; BLR, bone marrow-to-liver uptake ratio; SD, standard deviation.

**Table 2:** Correlation of PET/CT parameters of BM with T stage, N stage, and TNM stage.

		BM SUV		BLR	
		Value	*p*-Value	Value	*p*-Value
T stage	T1T2	1.67 ± 0.33	0.013	0.81 ± 0.15	0.002
	T3T4	1.83 ± 0.38		0.91 ± 0.20	
N stage	N0	1.67 ± 0.34	0.032	0.82 ± 0.17	0.023
	N1–N3	1.80 ± 0.38		0.88 ± 0.17	
TNM stage	Stage I–II	1.63 ± 0.31	0.006	0.80 ± 0.16	<0.001
	Stage III–IV	1.80 ± 0.38		0.89 ± 0.18	

**Table 3:** Univariate and multivariate analyses of disease progression-free survival.

Variables	Univariate Analysis		Multivariate Model with BM SUV		Multivariate Model with BLR	
	HR (95% CI)	*p*-Value	HR (95% CI)	*p*-Value	HR (95% CI)	*p*-Value
Age (<60 years versus ≥60 years)	0.97 (0.55–1.73)	0.929				
Sex (woman versus. man)	1.32 (0.59–2.95)	0.502				
Smoking (no versus yes)	1.03 (0.58–1.81)	0.932				
T stage (T1T2 versus T3T4)	1.77 (1.00–3.13)	0.048				
N stage (N0 versus N1–N3)	2.01 (1.13–3.58)	0.018				
TNM stage (stage I–II versus stage III–IV)	4.12 (2.05–8.30)	<0.001	2.98 (1.30–6.89)	0.010	2.87 (1.23–6.69)	0.014
Tumor size (<3.0 cm versus ≥3.0 cm)	2.23 (1.26–3.93)	0.006	1.29 (0.67–2.51)	0.447	1.17 (0.61–2.26)	0.632
CRP (<5.0 mg/dL versus ≥5.0 mg/dL)	1.70 (1.01–3.01)	0.047	1.32 (0.73–2.41)	0.357	1.34 (0.73–2.43)	0.342
NLR (<2.75 versus ≥2.75)	1.41 (0.80–2.50)	0.239				
PLR (<201.00 versus ≥201.00)	2.33 (1.30–4.20)	0.005	1.85 (1.02–3.37)	0.045	1.69 (0.92–3.11)	0.091
Maximum SUV (<6.60 versus ≥6.60)	3.98 (1.86–8.52)	<0.0011	2.65 (1.02–6.87)	0.045	2.38 (1.01–6.19)	0.046
MTV (<8.50 cm^3^ versus ≥8.50 cm^3^)	2.49 (1.39–4.48)	0.002				
TLG (<32.50 g versus ≥32.50 g)	2.26 (1.26–4.06)	0.007	1.52 (0.85–3.20)	0.124	1.59 (0.87–3.28)	0.179
BM SUV (<1.82 versus ≥1.82)	2.17 (1.23–3.83)	0.007	1.77 (0.95–3.28)	0.072		
BLR (<0.85 versus ≥0.85)	2.77 (1.50–5.12)	0.001			1.96 (1.01–3.80)	0.044

Legend: HR, hazard ratio; CI, confidence interval.

**Table 4:** Univariate and multivariate analyses of distant failure-free survival.

Variables	Univariate Analysis		Multivariate Analysis	
	HR (95% CI)	*p*-Value	HR (95% CI)	*p*-Value
Age (<60 yr versus ≥60 yr)	1.27 (0.48–3.35)	0.623		
Sex (woman versus man)	3.43 (0.46–25.88)	0.232		
Smoking (no versus yes)	1.06 (0.41–2.74)	0.911		
T stage (T1T2 versus T3T4)	2.34 (0.90–6.07)	0.081		
N stage (N0 versus N1–N3)	3.33 (1.17–9.47)	0.024		
TNM stage (stage I–II versus stage III–IV)	7.23 (1.65–31.72)	0.009	3.59 (0.61–21.33)	0.159
Tumor size (<3.0 cm versus ≥3.0 cm)	4.33 (1.60–11.72)	0.004	2.06 (0.62–6.83)	0.237
CRP (<5.0 mg/dL versus ≥5.0 mg/dL)	1.54 (0.94–2.70)	0.078		
NLR (<2.75 versus ≥2.75)	1.92 (0.74–4.99)	0.179		
PLR (<201.00 versus ≥201.00)	0.60 (0.17–2.08)	0417		
Maximum SUV (<6.60 versus ≥6.60)	5.45 (1.24–23.86)	0.024	1.39 (0.20–9.63)	0.740
MTV (<8.50 cm^3^ versus ≥8.50 cm^3^)	3.50 (1.23–9.95)	0.019		
TLG (<32.50 g versus ≥32.50 g)	4.29 (1.40–13.17)	0.011	0.92 (0.20–4.18)	0.916
BM SUV (<1.82 versus ≥1.82)	1.89 (0.73–4.91)	0.189		
BLR (<0.85 versus ≥0.85)	8.28 (1.89–36.24)	0.005	2.35 (0.73–7.61)	0.154

## References

[1] Lin W.W., Karin M. (2007). A cytokine-mediated link between innate immunity, inflammation, and cancer. J. Clin. Investig..

[2] Qu X., Tang Y., Hua S. (2018). Immunological Approaches Towards Cancer and Inflammation: A Cross Talk. Front. Immunol..

[3] Hanahan D., Weinberg R.A. (2011). Hallmarks of Cancer: The Next Generation. Cell.

[4] Ahbap E., Sakaci T., Kara E., Sahutoglu T., Koc Y., Basturk T., Sevinc M., Akgöl C., Kayalar A.O., Ucar Z.A. (2016). Neutrophil-to-lymphocyte ratio and platelet-tolymphocyte ratio in evaluation of inflammation in end-stage renal disease. Clin. Nephrol..

[5] Dolan R.D., McSorley S.T., Horgan P.G., Laird B., McMillan D.C. (2017). The role of the systemic inflammatory response in predicting outcomes in patients with advanced inoperable cancer: Systematic review and meta -analysis. Crit. Rev. Oncol..

[6] Mascarella M.A., Mannard E., Silva S.D., Zeitouni A., Wurzba S.D.S. (2018). Neutrophil-to-lymphocyte ratio in head and neck cancer prognosis: A systematic review and meta-analysis. Head Neck.

[7] Lu A., Li H., Zheng Y., Tang M., Li J., Wu H., Zhong W., Gao J., Ou N., Cai Y. (2017). Prognostic significance of neutrophil to lymphocyte ratio, lymphocyte to monocyte ratio, and platelet to lymphocyte ratio in patients with nasopharyngeal carcinoma. Biomed. Res. Int..

[8] Fang Y., Xu C., Wu P., Zhang L.H., Li D.W., Sun J.H., Li W.F., Liao Z.S. (2017). Prognostic role of C-reactive protein in patients with nasopharyngeal carcinoma: A meta-analysis and literature review. Medicine.

[9] Lee J.W., Choi J.S., Lyu J., Lee S.M. (2018). Prognostic significance of 18 F-fluorodeoxyglucose uptake of bone marrow measured on positron emission tomography in patients with small cell lung cancer. Lung Cancer.

[10] Lee J.W., Lee M.S., Chung I.K., Son M.W., Cho Y.S., Lee S.M. (2017). Clinical implication of FDG uptake of bone marrow on PET/CT in gastric cancer patients with surgical resection. World J. Gastroenterol..

[11] Lee J.W., Seo K.H., Kim E.S., Lee S.M. (2017). The role of (18) F-fluorodeoxyglucose uptake of bone marrow on PET/CT in predicting clinical outcomes in non-small cell lung cancer patients treated with chemoradiotherapy. Eur. Radiol..

[12] Kwon H.W., Becker A.K., Goo J.M., Cheon G.J. (2017). FDG whole-body PET/MRI in oncology: A systematic review. Nucl. Med. Mol. Imaging.

[13] Helsen N., Wyngaert T.V.D., Carp L., Stroobants S. (2018). FDG-PET/CT for treatment response assessment in head and neck squamous cell carcinoma: A systematic review and meta-analysis of diagnostic performance. Eur. J. Nucl. Med. Mol. Imaging.

[14] Bonomo P., Merlotti A., Olmetto E., Bianchi A., Desideri I., Bacigalupo A., Franco P., Franzese C., Orlandi E., Livi L. (2018). What is the prognostic impact of FDG PET in locally advanced head and neck squamous cell carcinoma treated with concomitant chemo-radiotherapy? A systematic review and meta-analysis. Eur. J. Nucl. Med. Mol. Imaging.

[15] Allegra E., Saita V., De Natale M., Marino N., Trapasso S., Tamburrini S., Alessio C., Ippolito M. (2017). Use of PET/CT to detect local and regional laryngeal cancer recurrence after surgery. Rep. Med. Imaging.

[16] Inoue K., Goto R., Okada K., Kinomura S., Fukuda H. (2009). A bone marrow F-18 FDG uptake exceeding the liver uptake may indicate bone marrow hyperactivity. Ann. Nucl. Med..

[17] Bural G.G., Torigian A.D., Chen W., Houseni M., Basu S., Alavi A. (2010). Increased 18F-FDG uptake within the reticuloendothelial system in patients with active lung cancer on PET imaging may indicate activation of the systemic immune response. Hell. J. Nucl. Med..

[18] Cicone F., Loose D., Deron P., Vermeersch H., Signore A., Van De Vyvere F., Scopinaro F., Van De Wiele C. (2008). Prognostic value of FDG uptake by the bone marrow in squamous cell carcinoma of the head and neck. Nucl. Med. Commun..

[19] Lee J.W., Lee S.C., Kim H.J., Lee S.M. (2017). Prognostic value of bone marrow (18) F-FDG uptake on PET/CT in lymphoma patients with negative bone marrow involvement. Hell. J. Nucl. Med..

[20] Lee J.W., Na J.O., Kang D.Y., Lee S.Y., Lee S.M. (2017). Prognostic significance of FDG uptake of bone marrow on PET/CT in patients with non-small-cell lung cancer after curative surgical resection. Clin. Lung Cancer.

[21] Lee J.W., Ahn T.S., Baek M.J. (2018). Fluorine-18-fluorodeoxyglucose uptake of bone marrow on PET/CT can predict prognosis in patients with colorectal cancer after curative surgical resection. Eur. J. Gastroenterol. Hepatol..

[22] Lee J.W., Jeon S., Mun S.T., Lee S.M. (2017). Prognostic Value of Fluorine-18 Fluorodeoxyglucose Uptake of Bone Marrow on Positron Emission Tomography/Computed Tomography for Prediction of Disease Progression in Cervical Cancer. Int. J. Gynecol. Cancer.

[23] Bang J.I., Yoon H.J., Kim B.S. (2018). Clinical utility of FDG uptake within reticuloendothelial system on F-18 FDG PET/CT for prediction of tumor recurrence in breast cancer. PLoS ONE.

[24] Jeong S.Y., Kim S.J., Pak K., Lee S.W., Ahn B.C., Lee J. (2018). Prognostic value of 18F-fluorodeoxyglucose bone marrow uptake in patients with solid tumors. Medicine (Baltimore).

[25] Murata Y., Kubota K., Yukihiro M., Ito K., Watanabe H., Shibuya H. (2006). Correlations between 18F-FDG uptake by bone marrow and hematological parameters: Measurements by PET/CT. Nucl. Med. Biol..

[26] Elstrom R.L., Tsai D.E., Vergilio J.A., Downs L.H., Alavi A., Schuster S.J. (2004). Enhanced marrow [18F]fluorodeoxyglucose uptake related to myeloid hyperplasia in Hodgkin’s lymphoma can simulate lymphoma involvement in marrow. Clin. Lymphoma.

[27] Elboğa U., Şahin E. (2017). Relationship between reticuloendothelial systems’ FDG uptake level and clinicopathological features in patient with invasive ductal breast cancer. La Radiol. Med..

[28] Katano A., Takahashi W., Yamashita H., Yamamoto K., Ando M., Yoshida M., Saito Y., Abe O., Nakagawa K. (2017). The impact of elevated C-reactive protein level on the prognosis for oro-hypopharynx cancer patients treated with radiotherapy. Sci. Rep..

[29] Kim D.Y., Kim I.S., Park S.G., Kim H., Choi Y.J., Seol Y.M. (2017). Prognostic value of posttreatment neutrophil–lymphocyte ratio in head and neck squamous cell carcinoma treated by chemoradiotherapy. Auris Nasus Larynx.

[30] Seban R.D., Robert C., Dercle L., Yeh R., Dunant A., Reuze S., Schernberg A., Sun R., Mignot F., Terroir M. (2019). Increased bone marrow SUVmax on 18F-FDG PET is associated with higher pelvic treatment failure in patients with cervical cancer treated by chemoradiotherapy and brachytherapy. OncoImmunology.

[31] Ozmen O., Koyuncu A., Koksal D., Tatci E., Alagoz E., Demirag F., Gokcek A., Arslan N. (2016). The potential value of volume-based quantitative PET parameters and increased bone marrow uptake for the prediction of survival in patients with malignant pleural mesothelioma. Nucl. Med. Commun..

[32] Urbanska A.M., Zhang X., Prakash S. (2015). Bioengineered Colorectal Cancer Drugs: Orally Delivered Anti-Inflammatory Agents. Cell Biophys..

[33] Verdoodt F., Dehlendorff C., Friis S., Kjaer S.K. (2018). Non-aspirin NSAID use and ovarian cancer mortality. Gynecol. Oncol..

[34] Wiegand S., Zimmermann A., Wilhelm T., Werner A.J. (2015). Survival after Distant Metastasis in Head and Neck Cancer. Anticancer. Res..

[35] Bonomi M., Patsias A., Posner M., Sikora A. (2014). The Role of Inflammation in Head and Neck Cancer. Results Probl. Cell Differ..

[36] Grivennikov S.I., Greten F.R., Karin M. (2010). Immunity, Inflammation, and Cancer. Cell.

[37] Panwar A., Interval E., Lydiatt W.M. (2017). Emergence of a novel staging system for oropharyngeal squamous cell carcinoma based on HPV status. Oncology.

